# Control of the growth of human breast cancer cells in culture by manipulation of arachidonate metabolism

**DOI:** 10.1186/1471-2407-7-138

**Published:** 2007-07-25

**Authors:** Rasha Hammamieh, Dena Sumaida, XiaoYan Zhang, Rina Das, Marti Jett

**Affiliations:** 1 Walter Reed Army Institute of Research, Silver Spring, MD, USA

## Abstract

**Background:**

Arachidonate metabolites are important regulators of human breast cancer cells. Production of bioactive lipids are frequently initiated by the enzyme phospholipase A2 which releases arachidonic acid (AA) that is rapidly metabolized by cyclooxygenases (COX) or lipoxygenases (LO) to other highly potent lipids.

**Methods:**

In this study we screened a number of inhibitors which blocked specific pathways of AA metabolism for their antiproliferative activity on MCF-7 wild type and MCF-7 ADR drug resistant breast cancer cells. The toxicity of these inhibitors was further tested on human bone marrow cell proliferation.

**Results:**

Inhibitors of LO pathways (specifically the 5-LO pathway) were most effective in blocking proliferation. Inhibitors of platelet activating factor, a byproduct of arachidonate release, were also effective antiproliferative agents. Curcumin, an inhibitor of both COX and LO pathways of eicosanoid metabolism, was 12-fold more effective in blocking proliferation of the MCF-7 ADR^s ^cells compared to MCF-7 wild type (WT) cells. These inhibitors that effectively blocked the proliferation of breast cancer cells showed varying degrees of toxicity to cultures of human bone marrow cells. We observed greater toxicity to bone marrow cells with inhibitors that interfere with the utilization of AA in contrast to those which block utilization of its downstream metabolites. MK-591, MK-886, PCA-4248, and AA-861 blocked proliferation of breast cancer cells but showed no toxicity to bone marrow cells.

**Conclusion:**

These inhibitors were effective in blocking the proliferation of breast cancer cells and may be potentially useful in human breast cancer therapy.

## Background

Epidemiologic investigations have suggested an association of dietary fat intake with breast cancer risk. Bioactive lipids generated from these fat metabolites are known to increase proliferation in cancer cells. Various studies have suggested dietary fat content, especially polyunsaturated fatty acids, promotes tumor growth by increasing synthesis of eicosanoids, particularly arachidonic acid (AA) products [1-4]. The possible role of AA derived eicosanoids as regulators of neoplastic cell growth is an area of significant interest in breast cancer biology.

Phospholipase A_2 _(PLA_2_) is the family of enzymes, which specifically hydrolyzes the 2-acyl position of glycerophospholipid. It has been reported that the concentration of PLA_2 _was elevated in the lungs, breasts, and the digestive organs of patients with malignant tumors and that the incidence and magnitude of the elevation increased with advanced cancer stage [5,6]. In our previous work with wild type (WT) and drug-resistant (MCF-7 ADR) MCF-7 cells, we observed PLA_2 _activity with specificity toward either linoleoyl or arachidonyl phosphatidylinositol [7]. PLA_2_'s are usually most efficient with polyunsaturated fatty acids in the SN-2 position, which result in the release of AA [7]. AA is metabolized through the cyclooxygenase pathway, which results in prostaglandin production or through the 5-lipoxygenase (5-LO) pathway, which results in the production of leukotriene [8]. Both prostaglandins and leukotrienes directly stimulate the growth of malignant cells [9-11].

Metabolism of exogenous AA by lipoxygenase or cyclooxygenase pathways produces a myriad of highly potent bioactive lipids which include leukotrienes, HPETEs, HETEs, and prostaglandins. Many of these metabolites have been shown to play a significant role in cancer cell growth. The arachidonate-derived eicosanoids PGE2, LTB4, and 5-, 12-, and 15-HETEs have been shown to be significantly higher in human breast cancer cells than control cells [12]. In Swiss 3T3 cells, stimulation of DNA synthesis occurs predominantly by activation of arachidonic acid release, followed by its oxidation to PGE2 and stimulation of adenylyl cyclase [13]. Metabolites of arachidonic acid and linoleic acid served as regulators of the EGF transduction system in Syrian hamster embryo fibroblasts [14,15]. Initiation of growth of human myeloblastic leukemia cells is dependent upon the increased formation of AA and its derivatives, formed primarily via the lipoxygenase pathway and the initiation of growth in these cells was followed by the rapid release of AA, HETEs and phospholipids into the culture medium [16]. The inhibitors of lipoxygenase and cyclooxygenase metabolism were shown to block proliferation in a human gastric cell line derived from a stomach tumor [17,18]. The consequent alteration in PKC, catalyzed by phospholipase(s) activity in endothelial cells, regulates the growth-dependent changes in AA release [19].

Avis et al. reported that exogenous addition of 5-HETE was found to stimulate lung cancer growth in vitro [20]. When selective antagonists were used to inhibit 5-lipoxygenase metabolism, significant growth reduction resulted in a number of lung cancer cell lines. Similarly, LTB4 and 12(R)-HETE significantly increased proliferation of two colon carcinoma cell lines, HT-29 and HCT-15 [10]. However, isomers of these two compounds such as LTB5 and 12(S)-HETE failed to affect the proliferation rate of these two cell lines. This demonstrates the importance of specificity in cancer cell proliferation. Epidemiological studies show that death rates from colon cancer decreased 40% for individuals who took aspirin (AA inhibitor) more than 16 times/month [21]. The use of inhibitors to manipulate AA pathways will help us better understand the function of elevated PLA2 levels in cancer cells, which may lead to the discovery of new anti-cancer drugs.

In the present study we have examined the effect of various inhibitors of arachidonic acid signaling pathways on growth of breast cancer cells, especially the drug resistant ones. It has been a challenge to treat drug resistant cancer patients effectively that have less toxicity. We show that inhibitors of the 5-LO pathway can block growth of breast cancer cells, especially the drug resistant MCF-7 ADR line very effectively. The toxicity of these inhibitors was then tested on the growth of bone marrow cells. The selection of some of these inhibitors might be an alternative treatment regimen for the breast cancer patients especially those that develop drug resistance during their course of treatment.

## Methods

### Materials

MK-591 was a gift from Merck-Frost, Pointe Claire, Quebec, Canada. All other inhibitors were purchased from BIOMOL Research Laboratories, Inc. The lipoxygenase inhibition assay was obtained from Cayman Chemical Company (Ann Arbor, MI).

### Cells

The human breast cancer MCF-7 Wild Type (WT) and its Adriamycin-Resistant, (MCF-7 ADR) cells were obtained from Kenneth Cowan (NCI, NIH Bethesda, MD), in accordance with the WRAIR human use protocol for established breast cancer cells. The cells were grown in Improved Modified Essential Medium (IMEM) containing 8% fetal bovine serum, 50,000 units/liter penicillin and 5,000 μg/l streptomycin at 37°C with 5% CO_2_.

Human bone marrow mononuclear cells were obtained from healthy volunteer donors arranged by Poietic Technologies Inc. (Gaithersburg, MD) and used for colony assays without any prior treatment. The stromal colonies were grown in IMDM-based Long Term Culture Media (LTCM) containing 25% horse serum (Hyclone) with 5% CO_2 _and 100% humidity at 37°C.

### Methyl (^3^H)-thymidine incorporation

Inhibition of proliferation in MCF-7 WT or MCF-7 ADR cultures was performed in 96-well plates. Cells were plated 15,000 cells/well in 0.2 ml IMEM culture fluid^1 ^and incubated overnight in triplicate. Inhibitors were added (50 μl) to achieve the indicated concentration and incubated for three days. During the last 18 hours, Methyl(^3^H)-thymidine (1 μCi/well) was added. The cells were trypsinized and harvested using Packard Unifilter System. The filter plates were dried in the air. Then, 40 μL of Packard Microscint 0 scintillation cocktail was added to each well and the filter plates were counted using Packard Top-count.

Use of inhibitors in cell culture: The inhibitors were dissolved in the appropriate solvents namely DMSO or ethanol as recommended by the manufacturer for each compound. The stock solutions of these compounds were at 100 mM for NDGA, MK886, MK591, PCA-4248, and at 200 mM for curcumin, AA881 and ketoconazole. Stock solutions were then diluted in the cell culture media to obtain the final concentration for each compound. Similar amounts of the appropriate solvent were added to the control cells.

### Lipoxygenase inhibition assay

Measurements of the lipoxygenase enzyme activity were carried out using a lipoxygenase inhibition assay kit. MCF-7 breast cancer cells were treated with each of the inhibitors at 20 μM concentrations. Each treatment was carried out in triplicate. Cells were lysed using a lysis buffer and the lipoxygenase activity was determined in the control and treated cells using the lipoxygenase assay kit obtained from Cayman Co (Ann Arbor, MI). Each assay was carried out in triplicate using the plate provided by the kit as instructed by the manufacturer. Cells lysates from control and treated cells were added to the plate followed by the substrate (linoleic acid) and then the chromogen. Absorbance intensities were determined at 500 nm using a plate reader.

### Colony Assay

Human bone marrow stroma colony studies were performed using human bone marrow mononuclear cells^2^. Cells (2 × 10^5 ^cells/well) were plated in 4-well plates with 5 ml LTCM and inhibitors were added on the second day. The media was changed with fresh media either with (continuous treatment) or without drugs (pulse) every week. The stroma colonies were stained on the second and fourth week of treatment with HEMA 3 (differential hematology stain, CMS). Colonies with diameters larger than 1 mm were counted and the size of the each colony was measured.

The hematopoietic progenitor colony assay was performed using Methylcellulose-based Colony Cocktail from Stem Cell Technologies, Inc. (HCC-4434) containing 30% Fetal Bovine Serum; 50 ng/ml rh Stem Cell Factor; 10 ng/ml rh GM-CSF; 10 ng/ml rh IL-3; and 3 units/ml rh Erythropoietin. Inhibitors were premixed with the cocktail (4.5 ml) and were added with the cells and plated in a 35 mm diameter gridded tissue culture plate (Nunc. Inc.). After a two week incubation period, the hematopoietic colonies were counted using an inverted phase microscope with 40× magnification. Using the standard criteria developed by Stem Cell Technologies Inc. (Atlas of Human Hematopoietic Colonies), the colonies were classified into categories that included: CFU-GM, BFU-E, CFU-E, CFU-Mix, and CFU-GEMM.

Both stroma colony and hematopoietic colony assay experiments were performed with four replicates and repeated at least three times with different lots of cells. Regardless of the lot of the cells, the same trends were observed.

### Hoechst staining

MCF-7 WT cells were plated overnight on an eight well chamber slide, pretreated with 20 μM MK886, Curcumin or NDGA and incubated for 48 hours. The cells were then fixed with paraformaldehyde and stained with 10 μM HOECHST 33258 dye for 30 minutes. Cells with bright, fragmented, condensed nuclei were identified as apoptotic cells.

### Statistical analysis

All experiments were performed as a replicate of three treatments and each assay was performed three independent times. Data were analyzed using analysis of variance (ANOVA). Student's t tests were used to determine the significance between mean group values compared to the control. A *P *value of < 0.05 was considered statistically significant. The standard deviation was calculated for each assay at each time point of the assay. Data were expressed as means ± S.D.

## Results

### Effect of Lipoxygenase/Cyclooxygenase inhibitors on breast cancer cell growth: and apoptosis

**Figure 1:**
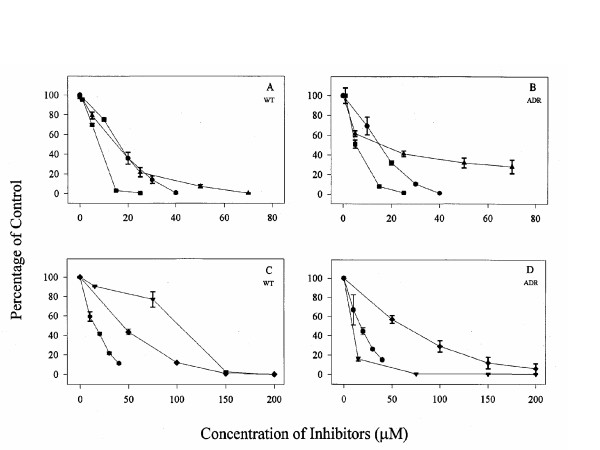
Inhibition of proliferation in (A and C) MCF-7 WT or (B and D) MCF-7 ADR cultures with lipoxygenase inhibitors; MK59I, MK886, NDGA for A and B; curcumin, ketoconazole, and AA88I for C and D. Cells were plated in IMEM culture fluid containing 8% FBS, 0.5% penicillin and streptomycin sulfate,0.5% kanamycin sulfate, and 1% MEM Vitamin Solution. Inhibitors were added after 24 hours and the cells were incubated in a humidified chamber of 5% CQ_2 _for an additional 48 hours. Thymidine was incorporated 16 hours prior to harvest and the cells were harvested using Packard Unifilter System. Values are expressed as % of the control. Data points represent the mean ± SD of three experiments with samples run in replicates of six.

Cells were plated in 96 well plates in the presence of various inhibitors of the arachidonic acid pathway and their growth was measured by 3H Thymidine incorporation. Inhibition of proliferation in MCF-7 WT or MCF-7 ADR cultures with lipoxygenase inhibitors is shown in figure 1. Inhibitors of lipoxygenase (LO) pathways were most effective at blocking proliferation. The effect of MK591, MK886 and NDGA was compared between the MCF-WT (Fig 1A) and MCF-7 ADR cells (Fig 1B). In the presence of these inhibitors, a concentration-dependent decrease in growth of these cells was observed. Curcumin, AA861, and ketoconazole also blocked growth of these breast cancer cells (Fig. 1C, D). Curcumin, a dual inhibitor of 5-lipoxygenase and cyclooxygenase and an agent with anti-inflammatory and anti-oxidant properties [22], was much more effective (ca. 11 fold) at blocking proliferation of the multi drug-resistant cells, MCF-7 ADR compared to MCF-7 wild type (WT) cells. It showed maximum inhibition of growth of breast cancer cells at very low concentrations.

Since metabolites of 5-LO have properties of a co-growth factor, it is possible that decreases in 5-LO products inhibit breast cancer cell proliferation. It is also possible that decreases in the ratio of lipoxygenase to cyclooxygenase products are responsible for the inhibition.

**Figure 2:**
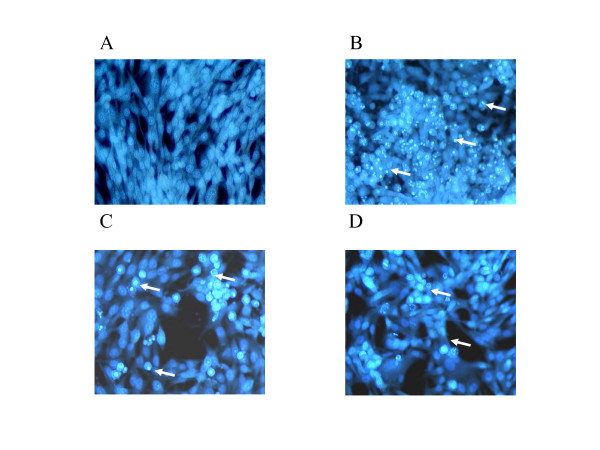
Induction of apoptosis in MCF-7 WT cells with (B) Curcumin, (C) NDGA and (D) MK 886. MCF-7 WT cells were plated in Labtek 8 chamber glass slides for 24 hours and then incubated with the inhibitors for 48 hours. Cells were fixed using paraformaldehyde and stained with HOECHST 33258 dye for 30 minutes. Cells with bright, fragmented, condensed nuclei were identified as apoptotic cells.

We studied the effect of curcumin, NDGA or MK886 on apoptosis in MCF-7 cells using the Hoechst staining method. As shown in figure 2, curcumin was the most effective in inducing apoptosis in these breast cancer cells after 24 hr.

### Effect of the inhibitors of the activity of 5-LO in MCF-7 cells

**Figure 3:**
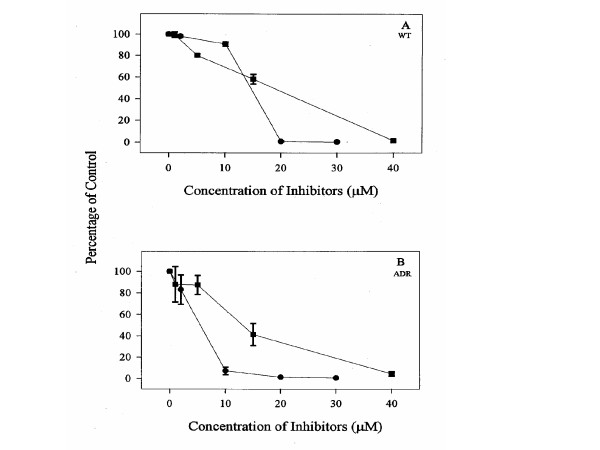
**Effect of the inhibitor on the activity of 5-LO in MCF-7**: Cells were treated with each inhibitor and the lipoxygenase activity was determined in treated and control cells. Data points represent the mean ± SD of three experiments with samples run in triplicate.

The lipoxygenase activity was studied using the Cayman lipoxygenase inhibitor screening assay that measures the hydroperoxides produced in the lipoxygenation reaction. MCF-7 WT Cells were treated with MK886, NDGA or Curcumin and the enzyme activity was determined in the control and inhibitor treated cells. As shown in figure 3, these compounds effectively inhibited the activity of 5-LO in MCF-7 cells. This correlates with the findings by Avis et al that showed that these inhibitors inhibited the activity of 5-LO in MCF-7 breast cancer cells [23].

### Regulation of breast cancer cell growth by PAF inhibitors

**Figure 4:**
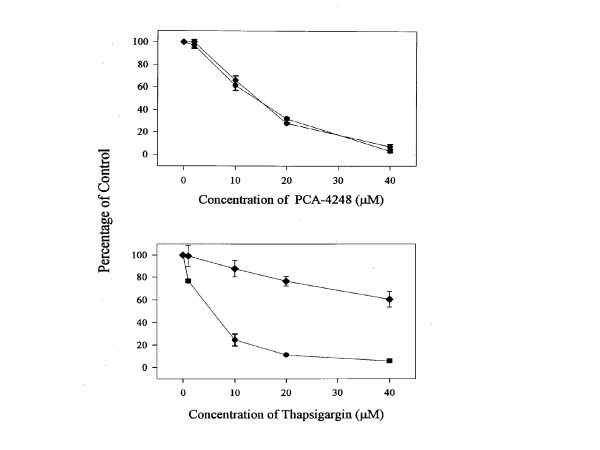
Inhibition of proliferation in MCF-7 WT and MCF-7 ADR cultures with PAP binding inhibitors (A) PCA-4248 and (B) Thapsigargin. Cells were harvested with the same procedure used in figure 2. Values are expressed as % of the control. Data points represent the mean ± SD of three independent experiments with samples run in replicates of six.

PCA-4248 inhibits PAF (a byproduct released from arachidonate metabolism) binding to human platelet and PMNL receptors. Inhibition of proliferation in MCF-7 WT or MCF-7 ADR cultures with PCA-4248 is shown in Figure 4. Blocking PAF binding results in the accumulation of arachidonate metabolism products, which may account for the inhibition of proliferation in MCF-7 WT or MCF-7 ADR cultures by PCA-4248.

### Human Bone Marrow progenitor colonies in the presence of inhibitors

**Figure 5:**
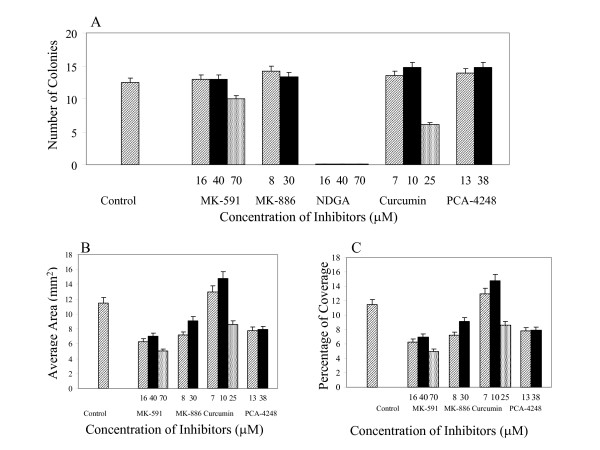
Human bone marrow stroma colonies treated with inhibitors for the first week and fresh media for the second week. (A) The number of colonies, (B) average area of the colonies, (C) and the percentage of coverage of the colonies are shown. The corresponding concentration of inhibitors were added to the cells which were plated in IMDM-based Long Term Culture Media containing 25% horse serum and incubated in a humidified chamber containing 5% CQ_2_. Data points represent the mean ± SD of three experiments.

**Figure 6:**
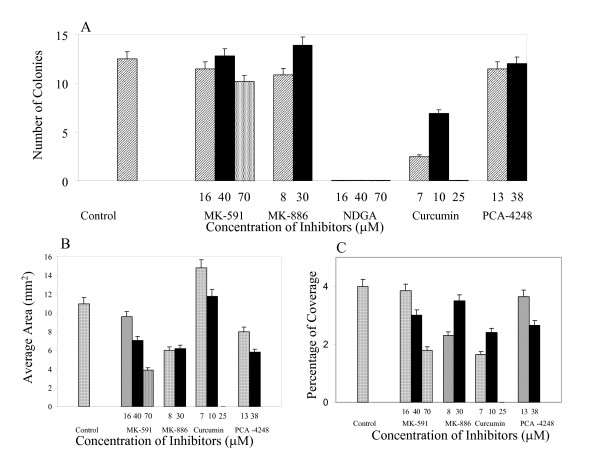
Human bone marrow stroma colonies treated with inhibitors continuously for 2 weeks. (A) The number of colonies, (B) average area of the colonies, (C) and the percentage of coverage of the colonies are shown. Cells were treated in the same manner as described in figure 5. Data points represent the mean ± SD of three independent experiments.

For any drug to be useful for chemotherapy it is important to study the toxicity of the drugs on bone marrow cells. To determine the toxicity of these inhibitors, the ability of human bone marrow cells to form colonies was studied. Human bone marrow stroma colonies with one week of treatment with the inhibitors are shown in Figure 5 (two week colonies). When compared to the control values, neither the total number of stroma colonies, the average area of each colony, nor the percentage coverage of the plate changed in the presence of MK-591, MK-886, and PCA-4248 at IC_50_. However, the stroma colonies were wiped out even with concentrations of the inhibitors lower than IC_50 _for NDGA. Lipoxygenase inhibitors (MK-591 and MK-886) did not have any toxic effect on the formation of stroma colonies. However, PAF Binding Inhibitor PCA-4248, did not affect the growth of stroma colonies when the concentration was at IC_90_. When the human bone marrow cells were treated for two weeks or four weeks continuously with the inhibitors, the growth of the colonies show similar trends to those treated for one week (Figure 6).

**Figure 7:**
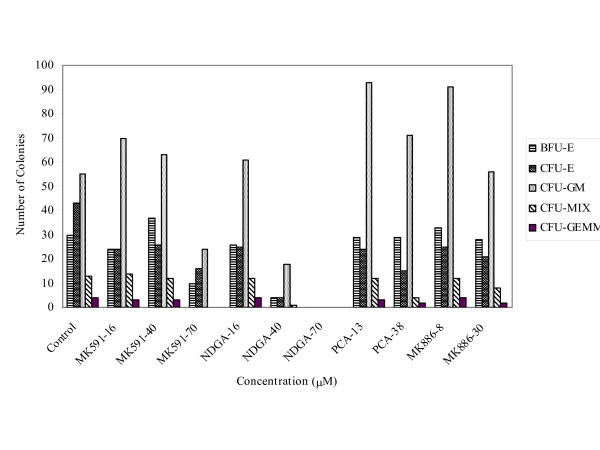
Hematopoietic colony distribution treated in cells with different concentrations of inhibitors. BFU-E (burst forming unit-erythroid) are progenitor cells with great proliferative capacity that give rise to large, multi-clustered erythroid colonies, CFU-E (colony-forming unit-erythroid) progenitors give rise to the smallest and most rapidly maturing erythroid colonies, CFU-GM (colony-forming unit-granulocyte, macrophage) are defined on the basis of their ability to produce colonies containing a minimum of either 20 or 50 cells, CFU-Mix (colony-forming unit-mixture) consists of CFU-E and CFU-GM combined colonies, and CFU-GEMM (colony-forming unit-granulocyte, erythroid, macrophage, megakaryocyte) are progenitor cells that give rise to colonies containing multiple lineages including erythroid cells.

Figure 7 shows the distribution of hematopoietic colonies categorized as BFU-E, CFU-E, CFU-GM, CFU-mix and CFU-GEMM in the presence of these inhibitors. Again MK-591, MK-886, and PCA-4248 showed no toxicity on the hematopoietic colonies since the relative population of the hematopoietic colonies in these treated samples was similar to the control. NDGA was toxic to hematopoietic colonies only at higher concentrations. Thus the MK drugs and PCA are some of the drugs that may have some use in the clinics in future because they are safe and effective in blocking the growth of breast cancer cells.

## Discussion

There is a constant need for additional therapeutic strategies to combat the disease in order to improve the quality of life and prolong survival for women with breast cancer. Bioactive lipids play an important role in the growth and development of the normal mammary gland. Understanding the regulatory role of these lipids on the control of the epithelial cell population is extremely important. Dietary fat content, especially polyunsaturated fatty acids, promotes tumor growth by increasing synthesis of eicosanoids particularly AA products [24]. The possible role of arachidonic acid derived eicosanoids as regulators of neoplastic cell growth is an area of significant interest in breast cancer biology.

Signal transduction explains the molecular events by which extracellular signals elicits an intracellular response. The generation of a variety of lipid signal transduction molecules from hydrolysis of membrane phospholipids is an early response. Several types of signal transduction pathway inhibitors effectively blocked proliferation in MCF-7 WT or MCF-7 ADR cultures. Lipoxygenase (LO) inhibitors NDGA, MK591, AA-861 and MK886 were equally effective at blocking proliferation for MCF-7 WT and MCF-7 ADR cells. We confirmed the inhibition of 5-LO enzyme activity in MCF-7 by the inhibitors tested at the concentrations used in the proliferation assays.

Curcumin has been shown to strongly inhibit the LO pathway and weakly inhibit the COX pathway. Curcumin inhibits proliferation by blocking the action of the Thymidine kinase enzyme that is necessary for cell cycle progression through the S-phase [25]. In immortalized NIH 3T3 and malignant cancer cell lines, Curcumin induced cell shrinkage, chromatin condensation, DNA fragmentation, and characteristics of apoptosis. In the present study, it inhibits the proliferation for both MCF-7 WT and MCF-7 ADR cells. However, the IC_50 _for MCF-7 ADR cells (12 μM) is much smaller than that for MCF-7 WT cells (90 μM). Interestingly, Curcumin effectively blocked proliferation in only MCF-7 ADR much more than in MCF-7 WT cells. However Ketoconazole was more effective with MCF-7 WT cells than MCF-7 ADR cells.

The cyclooxygenase (COX) enzyme is an important enzyme because it catalyzes the initial reaction of arachidonate metabolism that leads to the formation of prostaglandins, thromboxane, and prostacyclin. Recently, a second form of the cyclooxygenase (COX) enzyme, COX-2, has been isolated. A single amino acid difference in the active site (valine 509 to isoleucine) and a series of differences in the active site confers selectivity for COX-2. COX-2, which can be induced by cytokines and growth factors, is linked to inflammatory cell types and tissues whereas COX-1's course of action resides primarily in the stomach and kidney [26,27]. It is possible that a selective COX-2 inhibitor may eliminate the side effects associated with COX-1 while still maintaining COX-2 inhibition and vice versa. Teh at al. has reported that Cox-2 inhibitors induced apoptosis and inhibited proliferation in MCF-7 cells [35].

An inhibitor of PAF (PCA-4248) effectively blocked proliferation of MCF-7 WT and MCF-7 ADR. Inhibitors of the LO pathway or specifically the 5-LO pathway were most effective at blocking proliferation. Blocking both LO and COX pathways in WT cells using Curcumin also inhibits proliferation. We have previously observed that AA metabolism in the presence of aspirin was shifted to large increases in LO products [20]. This might have occurred in the presence of these dual inhibitors, which could explain their lack of inhibitory activity.

NDGA, MK-591, and MK-886 blocked arachidonic acid metabolism pathway at different stages and all effectively inhibit the proliferation of MCF-7 WT and MCF-7 ADR cultures. MK-591 and MK-886 did not exhibit any toxicity on either human bone marrow stroma colonies or on human hematopoietic colonies at a concentration lower than IC_90_. However, NDGA completely blocked stroma colony formation even though the hematopoietic colonies still survived at a concentration lower than IC_70_. This could be due to the fact that it blocks the initial conversion from AA to any/all of the lipoxygenases and their products. PCA-4248, an inhibitor of PAF, did not have an effect on either stroma colonies or hematopoietic colonies at a concentration below IC_90_. Our results indicate that MK-591, MK-886, and PCA-4248 could be good candidates for medical clinical trials.

## Conclusion

Use of these inhibitors of different signaling pathways gives us a better understanding of the mechanism of action of the bioactive lipids in breast cancer. By blocking one of these pathways we were able to block the growth of breast cancer cells, especially the cells that develop drug resistance. However not all of these inhibitors serve as a potential therapeutic agents because of their toxicity to the bone marrow cells. The MK drugs, which block 5-LO pathway and block cell growth, were not toxic to the bone marrow cells and therefore may be of some use in controlling the growth of breast cancer cells opening new avenues for therapeutic intervention.

## Abbreviations

AA: Arachidonic acid

COX: Cyclooxygenases

LO: Lipoxygenases

PLA_2_: Phospholipase A_2_

PGE2: Prostaglandin E2

LTB4: Leukoriene B4

HETE: Hydroxyeicosatetraenoic acid

## Competing interests

The author(s) declare that they have no competing interests.

## Authors' contributions

RH drafted the manuscript, performed the statistical analysis and participated in the proliferation and apoptosis studies.

DS carried out cell cultures, treatments with the inhibitors and the lipoxygenase assay.

XZ participated in the proliferation and bone marrow assays.

RD participated in the design on the study and in drafting the manuscript.

MJ conceived of the study, and participated in its design and coordination.

All authors read and approved the final manuscript.
